# RNAseq based variant dataset in a black poplar association panel

**DOI:** 10.1186/s13104-023-06521-w

**Published:** 2023-10-02

**Authors:** Odile Rogier, Aurélien Chateigner, Marie-Claude Lesage-Descauses, Claire Mandin, Véronique Brunaud, José Caius, Ludivine Soubigou-Taconnat, José Almeida-Falcon, Catherine Bastien, Vanina Benoit, Guillaume Bodineau, Nathalie Boizot, Corinne Buret, Jean-Paul Charpentier, Annabelle Déjardin, Alain Delaunay, Régis Fichot, Véronique Laine Prade, Françoise Laurans, Isabelle Le Jan, Anne-Laure Legac, Stéphane Maury, Mesfin Nigussie Gebreselassie, Patrick Poursat, Céline Ridel, Léopoldo Sanchez, Véronique Jorge, Vincent Segura

**Affiliations:** 1 INRAE, ONF, BioForA, 45075 Orléans, France; 2 Université Paris-Saclay, CNRS, INRAE, Université Evry, Institute of Plant Sciences Paris-Saclay (IPS2), 91190 Gif sur Yvette, France; 3 Université Paris Cité, CNRS, INRAE, Institute of Plant Sciences Paris-Saclay (IPS2), 91190 Gif sur Yvette, France; 4 Groupe d’Étude et de Contrôle des Variétés et des Semences (GEVES), Station Nationale d’Essais de Semences (SNES), 49071 Beaucouzé, France; 5 INRAE, InfoSol, 45075 Orléans, France; 6 INRAE, GBFor, 45075 Orléans, France; 7 Laboratoire de Biologie des Ligneux et des Grandes Cultures, INRAE, EA1207 USC1328, Université d’Orléans, 45067 Orléans, France; 8 UMR AGAP Institut, CIRAD, INRAE, Institut Agro Montpellier, Univ Montpellier, 34398 Montpellier, France

**Keywords:** *Populus nigra*, RNA polymorphisms, SNV discovery, Genotyping, Bioinformatics pipeline, Transcriptomics

## Abstract

**Objective:**

Black poplar (*Populus nigra* L.) is a species native to Eurasia with a wide distribution area. It is an ecologically important species from riparian ecosystems, that is used as a parent of interspecific (*P. deltoides* x *P. nigra*) cultivated poplar hybrids. Variant detection from transcriptomics sequences of 241 *P. nigra* individuals, sampled in natural populations from 11 river catchments (in four European countries) is described here. These data provide new valuable resources for population structure analysis, population genomics and genome-wide association studies.

**Data description:**

We generated transcriptomics data from a mixture of young differentiating xylem and cambium tissues of 480 *Populus nigra* trees sampled in a common garden experiment located at Orléans (France), corresponding to 241 genotypes (2 clonal replicates per genotype, at maximum) by using RNAseq technology. We launched on the resulting sequences an in-silico pipeline that allowed us to obtain 878,957 biallelic polymorphisms without missing data. More than 99% of these positions are annotated and 98.8% are located on the 19 chromosomes of the *P. trichocarpa* reference genome. The raw RNAseq sequences are available at the NCBI Sequence Read Archive SPR188754 and the variant dataset at the *Recherche Data Gouv* repository under https://doi.org/10.15454/8DQXK5.

## Objective

Of the twenty-nine species in the genus *Populus*, black poplar (*P. nigra* L.) is native to Eurasia with a wide distribution area including Europe, as well as the southwest and central Asia, and northwest Africa [1]. It is regarded as a keystone species for riparian ecosystems in ecological and conservation studies [2] and it has an interest as a parental pool in interspecific (*P. deltoides* x *P. nigra*) poplar breeding programs as the origin of cultivated hybrids [3].

In this study, we selected 241 genotypes from a *P. nigra* collection of 587 genotypes previously genotyped with an Illumina 12 K Infinium Bead-Chip array (8000 Single Nucleotide Variants (SNVs); [4]), which in turn belong to a larger collection of 1098 cloned genotypes sampled in natural populations from 11 river catchments [5] in four European countries. These 241 genotypes were previously studied for wood properties in 2 sites (Savigliano-2011 & Orléans-2012; [6]), and their selection was based on the following set of criteria defined following a first analysis of population structure with 8000 SNVs: (i) introgression < 10% of the worldwide-spread fastigiated form *P. nigra* var *italica*, (ii) proportion of recruitment to their ancestral population > 50% and (iii) survival in the common garden located at Orléans, France.

We generated transcriptomics data from young differentiating xylem and cambium tissues of these *P. nigra* selected genotypes by using RNAseq technology. We launched on the resulting sequences an in-silico pipeline [7] that allowed us to obtain 878,957 polymorphisms. We already used this data in Chateigner et al. [8] and in Wade et al. [9] for phenotype prediction. These data provide new valuable resources for a wide variety of genome-based studies, ranging from population structure analysis over distribution ranges, to genomic prediction and genome-wide association studies (GWAS) for traits related to wood properties and growth, for example.

## Data description

Young differentiating xylem and cambium tissues were harvested in June 2015 from 480 *Populus nigra* trees from a common garden located at Orléans (France) (241 genotypes, 2 clonal trees per genotype, Data file 1 [10], Data set 1 [11]). RNA from the xylem and cambium were extracted with RNeasy Plant kit (Qiagen, France) according to manufacturer’s recommendations. Treatment with DNase I (Qiagen, France) was carried out to ensure elimination of genomic DNA. RNA was eluted in RNAse-DNAse free water and quantified with a Nanodrop spectrophotometer. RNA from xylem and cambium of the same plant were pooled in an equimolar extract (250 ng/μL) and sent to the sequencing platform. The sequencing platform POPS (transcriptOmic Platform of Institute of Plant Sciences—Paris-Saclay) prepared the RNAseq libraries from polyA-RNA selection using the TruSeq_Stranded_mRNA_SamplePrep_Guide_15031047_D protocol (Illumina, California, U.S.A.).

Identity of each sample was checked with the following procedure: a first round of variant detection was performed with FreeBayes (v.1.0.0) [12], then IBS (Identity By State) was calculated between samples of the same individual, as well as between the samples of the present study and the 852 individuals that had been previously genotyped with the Illumina 12 K Infinium Bead-Chip array [4] (Data file 2 [13]). After removing sampling errors and correcting identities, 241 unique genotypes were considered corresponding to 461 FASTQ files (Data set 2 [14]).

The sequences for each sample were processed with the pipeline defined in Rogier et al. [7] with small modifications to detect SNVs. All experiment steps (from growth conditions to bioinformatic analyses) are available in the CATdb database (Data set 3 [15]). Briefly, the reads were first trimmed with Trimmomatic (v.0.38) [16] to remove adapter and low-quality sequences. Then, they were aligned to the *Populus trichocarpa* reference genome v.3.0 [17] using the BWA-MEM algorithm (v.0.7.12) [18]. We followed the GATK Best Practices [19, 20] for RNAseq short variant discovery: we first marked the duplicates with the MarkDuplicates from the Picard tools (v.2.0.1) [21] and then used the SplitNCigarReads, the Indel Realignment and the Base Quality Recalibration tools from GATK (v.3.5) [22]. SNV and short insertions and deletions were genotyped for all the sequenced trees (the same genotypes were pooled together) with 3 variants callers: (i) GATK using the HaplotypeCaller tool in single-sample calling mode followed by joint genotyping of the samples with the GenotypeGVCFs tool; (ii) FreeBayes (v.1.0.0) [12] in a multi-sample mode and (iii) the mpileup tool from SAMtools (v.1.3.1) [23] in a multi-sample mode followed by bcftools (v.1.3.1) [24].

The resulting 3 files (one per caller) were filtered with VCFtools (v.0.1.15) [25] to obtain only the intra-specific (*Populus nigra*) biallelic SNV with a variant quality score (QUAL) threshold over 30. These filtered files were then combined together with the vcf-isec tool from VCFtools and we only kept the genotype calls that were detected by at least 2 variant callers. Otherwise, the genotype call was set as a missing value for this particular individual. As there remained some missing data (Data file 3 [26]), genotype imputation was performed using the Fimpute program (v.2.2) [27] for the SNV located on the chromosomes/scaffolds that contain at least 2 SNV. Thereby, 9.73% of missing values were imputed. This yielded genotypes at 878,957 biallelic sites for the 241 *Populus nigra* individuals (Data file 4 [28]) without missing data. 878,893 of them have an annotation (Data file 5 [29], Data file 6 [30]) from ANNOVAR (v.2017Jul16) [31] (Data file 7 [32]). Among them, 868,861 are located on the 19 chromosomes (Data file 7, [32]). A total of 26,909 genes harbored between 1 and 309 SNVs.

## Limitations

- The SNVs are limited to expressed genes: 26,909 vs a maximum of 41,335 *P. trichocarpa* protein-coding genes. We found a correlation between expression level and SNV density at gene level, which is likely due to an increase in coverage for highly expressed genes (Data file 7 [32]).
- RNA were extracted from young differentiating xylem and cambium tissues, which might not be representative of transcriptional activity of other tissues of the tree. The found SNVs are therefore specific to the genes expressed in these two tissues. Nevertheless, this makes this SNV dataset clearly appropriate to study the genetics of wood formation.
- The SNVs are limited to the gene space: 92.55% of the found SNV are exonic, intronic, in the 3’UTR (UnTranslated Region) or in the 5’UTR (Data file 7 [32]).

## Data Availability

The raw RNAseq sequences are available in the National Center for Biotechnology Information (NCBI) Sequence Read Archive with the SPR188754 identifier [14]. The 241 associated accessions can be found in the GnpIS Information System under the collection “POPULUS_NIGRA_RNASEQ_PANEL” [11] or with the DOIs in the table of the studied genotypes [10]. The steps of the experiment (from growth conditions to bioinformatic analyses) are described in the CATdb database [15]. The other data described in this Data note can be freely and openly accessed on the *Recherche Data Gouv* repository under https://doi.org/10.15454/8DQXK5 [33]. Please see Table

**Table 1:** Overview of data files/data sets

Label	Name of data file/data set	File types (file extension)	Data repository and identifier (DOI or accession number)
Data file 1	collection_POPULUS_NIGRA_RNASEQ_PANEL *(list of the studied genotypes)*	Tabular text file (.tab)	*Recherche Data Gouv* repository: https://doi.org/10.57745/GKXDSQ; [10]
Data set 1	Information on the studied genotypes	Web files (.html)	GnpIS: collection:POPULUS_NIGRA_RNASEQ_PANEL; [11]
Data file 2	SNV_POPULUS_NIGRA_RNASEQ_PANEL_quality_control *(Quality control figures)*	PDF file (.pdf)	*Recherche Data Gouv* repository: https://doi.org/10.57745/SSDFV2; [13]
Data set 2	Raw RNA-seq files	Sequencing files (.fastq)	NCBI Sequence Read Archive: SPR188754; [14]
Data set 3	Steps of the experiment, from growth conditions to bioinformatic analyses	Web files (.html)	CATdb: experiment = 640; [15]
Data file 3	SNV_raw_POPULUS_NIGRA_RNASEQ_PANEL (*Initial variant calling file*)	Variant Call Format file (.vcf)	*Recherche Data Gouv* repository: https://doi.org/10.57745/RBR6X0; [26]
Data file 4	SNV_imputated_POPULUS_NIGRA_RNASEQ_PANEL (*Imputated variant calling file*)	Variant Call Format file (.vcf)	*Recherche Data Gouv* repository: https://doi.org/10.57745/5IQLI9; [28]
Data file 5	SNV_imputated_POPULUS_NIGRA_RNASEQ_PANEL.variant_function (*Annotation file1*)	Tabular text files (.tab)	*Recherche Data Gouv* repository: https://doi.org/10.57745/PAEKL7; [29]
Data file 6	SNV_imputated_POPULUS_NIGRA_RNASEQ_PANEL.exonic_variant_function (*Annotation file2*)	Tabular text files (.tab)	*Recherche Data Gouv* repository: https://doi.org/10.57745/EG9HOE; [30]
Data file 7	SNV_imputated_POPULUS_NIGRA_RNASEQ_PANEL_analysis_figures (*Analysis figures*)	PDF file (.pdf)	*Recherche Data Gouv* repository: https://doi.org/10.57745/BTBR; [32]

1 and references [13, 26, 28–30, 32] for details and links to the data. Overview of data files/data sets GnpIS: collection:POPULUS_NIGRA_RNASEQ_PANEL; [11]

## Abbreviations

GATK: Genome analysis ToolKit
GWAS: Genome-wide association studies
IBS: Identity by state
mRNA: Messenger RiboNucleic Acid
NCBI: National center for biotechnology information
RNA: RiboNucleic acid
RNAseq: RiboNucleic acid sequencing
SNV: Single nucleotide variant
UTR: UnTranslated region
